# Supplementary material for: Subordinate Effect of -21M HLA-B Dimorphism on NK Cell Repertoire Diversity and Function in HIV-1 Infected Individuals of African Origin
Source: Front Immunol. 2020 Feb 18;11:156. doi: 10.3389/fimmu.2020.00156 (PMC7041644; doi:10.3389/fimmu.2020.00156)
Supplement: Supplementary file 1 [file Table_1.pdf]

Table S1. Subject characteristics and HLA genenotypes

Subjects	HLA-B -21	SEX	Age	Country	Plasma HIV Vl	HLA-B allele 1	HLA-B allele 1	HLA-B allele 2	HLA-B allele 2	HLA-C allele 1	HLA-C allele 1	HLA-C allele 2	HLA-C allele 2	Bw4/Bw6
01	M/M	F	26	Malawi	4.28712962	*08	*0801g	*14	*1401	*02	*0210	*16	*1601	Bw6/Bw6
2	M/M	F	21	Malawi	4.27671449	*42	*4201	*42	*4202	*17	*1701	*17	*1701	Bw6/Bw6
3	M/M	F	29	Malawi	4.59584881	*07	*0702	*81	*8101g	*07	*0702g	*18	*1801g	Bw6/Bw6
4	M/M	F	22	South Africa	4.06818586	*14	*1402	*42	*4202	*08	*0802	*17	*1701	Bw6/Bw6
5	M/M	F	31	South Africa	2.65030752	*81	*8101g	*07	*0702g	*07	*0702g	*18	*1801g	Bw6/Bw6
6	M/M	F	43	Tanzania	3.86628734	*14	*1401	*42	*420101	*08	*080201	*17	*1701/02/03	Bw6/Bw6
7	M/M	F	50	Tanzania	4.27902781	*07	*0702/44/49	*42	*420101	*07	*0702/50/66	*17	*1701/02/03	Bw4/Bw6
8	M/M	F	32	Tanzania	5.31964105	*42	*42:02	*48	*48:05	*17	*17:01	*15	*15:05/09/2	Bw6/Bw6
9	M/M	F	22	South Africa	4.45334892	*07	*0702g	*14	*1402	*07	*0702g	*08	*0802	Bw6/Bw6
10	M/M	F	23	South Africa	2.60205999	*81	*8101/02	*07	*0703/16/37	*18	*1801/02	*04	*0407/*180	Bw6/Bw6
11	M/M	F	21	Malawi	4.37235958	*07	*0703	*67	*6701	*07	*0702g	*12	*1203	Bw6/Bw6
12	T/T	F	22	Malawi	4.70854837	*15	*1503g	*35	*3501/28	*02	*0210	*04	*0401g	Bw6/Bw6
13	T/T	F	31	Malawi	4.23739292	*41	*4101	*45	*4501g	*06	*0602	*17	*1701	Bw6/Bw6
14	T/T	F	19	South Africa	5.2380461	*15	*1503g	*45	*4501g	*02	*0210	*06	*0602	Bw6/Bw6
15	T/T	F	22	South Africa	4.45939249	*18	*1801g	*35	*3501g	*04	*04:01/09N/	*07	*07:04/11	Bw6/Bw6
16	T/T	F	39	South Africa	3.69635639	*15	*1510	*15	*1510	*03	*0304	*04	*0401g	Bw6/Bw6
17	T/T	F	22	South Africa	3.82197182	*15	*151001	*18	*1801/17N	*07	*0704/11	*08	*0804	Bw6/Bw6
18	T/T	F	35	Tanzania	3.78561452	*15	*1503/103	*45	*4501/07	*07	*0701/06/18	*16	*160101	Bw6/Bw6
19	T/T	F	20	Malawi	4.24519176	*45	*4501/07	*57	*570301	*16	*160101	*18	*1801/02	Bw4/Bw6
20	T/T	F	35	Malawi	4.4798775	*44	*4403	*45	*4501g	*14	*1403	*16	*1601	Bw4/Bw6
21	T/T	F	29	Tanzania	5.45947693	*18	*18:01/53/5	*53	*53:01	*04	*04:01/09N/	*07	*07:04/11	Bw4/Bw6
22	T/T	F	23	Malawi	4.46097297	*18	*18:01/53/5	*53	*53:01	*04	*04:01/09N/	*07	*07:04/11	Bw4/Bw6
23	T/T	F	28	Malawi	4.67858211	*15	*15:03/61/1	*41	*41:02/11	*17	*17:01	*02	*02:10	Bw6/Bw6
24	T/T	F	27	South Africa	2.60205999	*41	*4102/04	*15	*1503/61/74	*02	*0202/04/07	*02	*0203/16/*1	Bw6/Bw6
25	M/T	F	34	Malawi	4.25558605	*15	*1503g	*42	*4201	*02	*0210	*17	*1701	Bw6/Bw6
26	M/T	F	22	Malawi	4.64674671	*08	*0801g	*15	*1510	*03	*0304	*07	*0701g	Bw6/Bw6
27	M/T	F	31	Malawi	6.01989483	*14	*1402	*18	*1801g	*07	*0704/12	*08	*0802	Bw6/Bw6
28	M/T	F	24	Malawi	5.1033384	*07	*07:02/44/4	*18	*18:01/53/5	*07	*07:04/11/1	*07	*07:02/50/6	Bw6/Bw6
29	M/T	F	30	South Africa	4.30749604	*15	*1510	*39	*3910	*08	*0804	*12	*1203	Bw6/Bw6
30	M/T	F	22	South Africa	3.94101424	*08	*0801g	*15	*1510	*04	*0401g	*07	*0701g	Bw6/Bw6
31	M/T	F	21	South Africa	3.73965144	*15	*151001	*08	*0801/08N/1	*07	*0701/06/18	*08	*0804	Bw6/Bw6
32	M/T	F	38	South Africa	3.15594302	*08	*0801g	*15	*1510	*03	*0304	*03	*0304	Bw6/Bw6
33	M/T	F	53	Tanzania	3.59117595	*15	*1503/103	*39	*3924	*02	*0210	*07	*0701/06/18	Bw6/Bw6
34	M/T	F	27	Malawi	5.17000047	*15	*1503g	*42	*4201	*02	*0210	*17	*1701	Bw6/Bw6
35	M/T	F	26	Malawi	4.85868755	*15	*151001	*42	*4202	*03	*030402	*17	*1701/02/03	Bw6/Bw6
36	M/T	F	26	Malawi	4.98582152	*14	*14:02/20/2	*15	*15:10	*04	*04:01/09N/	*16	*16:01/32/3	Bw6/Bw6
