# Supplementary material for: Subordinate Effect of -21M HLA-B Dimorphism on NK Cell Repertoire Diversity and Function in HIV-1 Infected Individuals of African Origin
Source: Front Immunol. 2020 Feb 18;11:156. doi: 10.3389/fimmu.2020.00156 (PMC7041644; doi:10.3389/fimmu.2020.00156)
Supplement: Supplementary file 2 [file Table_2.pdf]

Table S2. Antibody List

Antibodies	Clone	Titration	Cat number	Company
Brilliant Violet 510 anti-human CD14	**M5E2**	1 in 100	**301842**	BioLegend
Brilliant Violet 510 anti-human CD19	**HIB19**	1 in 100	**302242**	BioLegend
PE/Dazzle 594 anti-human CD56 (NCAM)	**HCD56**	2 in 100	**318348**	BioLegend
Brilliant Violet 605 anti-human CD56 (NCAM)	**HCD56**	2 in 100	**318334**	BioLegend
Brilliant Violet 650 anti-human CD3	**OKT3**	1 in 100	**317324**	BioLegend
PerCP anti-human CD16	**3G8**	1 in 100	**302030**	BioLegend
Brilliant Violet 711 anti-human CD16	**3G8**	1 in 100	**302044**	BioLegend
PE anti-human HLA-E	**3D12**	1 in 100	**342604**	BioLegend
CD4 monoclonal antibody, APC-eFluor 780	**RPA-T4**	1 in 200	**47-0049-42**	eBiosciences
CD8a monoclonal antibody, Alexa Fluor 700	**OKT8**	1 in 200	**56-0086-42**	eBiosciences
PE anti-human CD328 (Siglec-7)	**6-434**	1 in 100	**339204**	Biolegend
PE mouse anti-human HLA-C	**DT-9**	1 in 200	**566372**	BD Biosciences
PeCy7 CD159a (NKG2a)	**Z199**	1 in 100	**B10246**	Beckman Coulter
APC KIR2DL2/2DL3/2DS2 (CD158b1/b2,j)	**GL183**	2 in 100	**A22333**	Beckman Coulter
PE anti-human NKG2C/CD159c	**134591**	4 in 100	**FAB138P**	R&D systems
Alexa Fluor® 700 anti-human NKG2C/CD159c	**134591**	5 in 100	**FAB138N**	R&D systems
APC anti-human KIR2DL1/KIR2DS5	**143211**	4 in 100	**FAB1844A**	R&D systems
APC anti-human KIR3DL2/CD158k	**539304**	2 in 100	**FAB2878A**	R&D systems
Brilliant Violet 421 mouse anti-human CD57	**NK-1**	1 in 200	**563896**	BD Biosciences
FITC mouse anti-human CD57	**NK-1**	2 in 100	**555619**	BD Biosciences
APC mouse anti-human CD158e (KIR3DL1)	**DX9**	2 in 100	**130-092-474**	Miltenyi Biotec
Brilliant Violet 421 mouse anti-human IFN-γ	**B27**	1 in 100	**562988**	BD Biosciences
Milli-Mark anti-FcεRI antibody, γ subunit-FITC	**Polyclonal**	Dilute primary Ab 1 in 5 in PBS; 3 in 100	**FCABS400F**	MerckMillipore
PE-CF594 mouse anti-human PLZF	**R17-809**	1 in 200	**565738**	BD Biosciences
APC-H7 mouse anti-human CD107a	**H4A3**	2 in culture	**561343**	BD Biosciences
