# Supplementary material for: Subordinate Effect of -21M HLA-B Dimorphism on NK Cell Repertoire Diversity and Function in HIV-1 Infected Individuals of African Origin
Source: Front Immunol. 2020 Feb 18;11:156. doi: 10.3389/fimmu.2020.00156 (PMC7041644; doi:10.3389/fimmu.2020.00156)
Supplement: Supplementary file 3 [file Image_1.pdf]

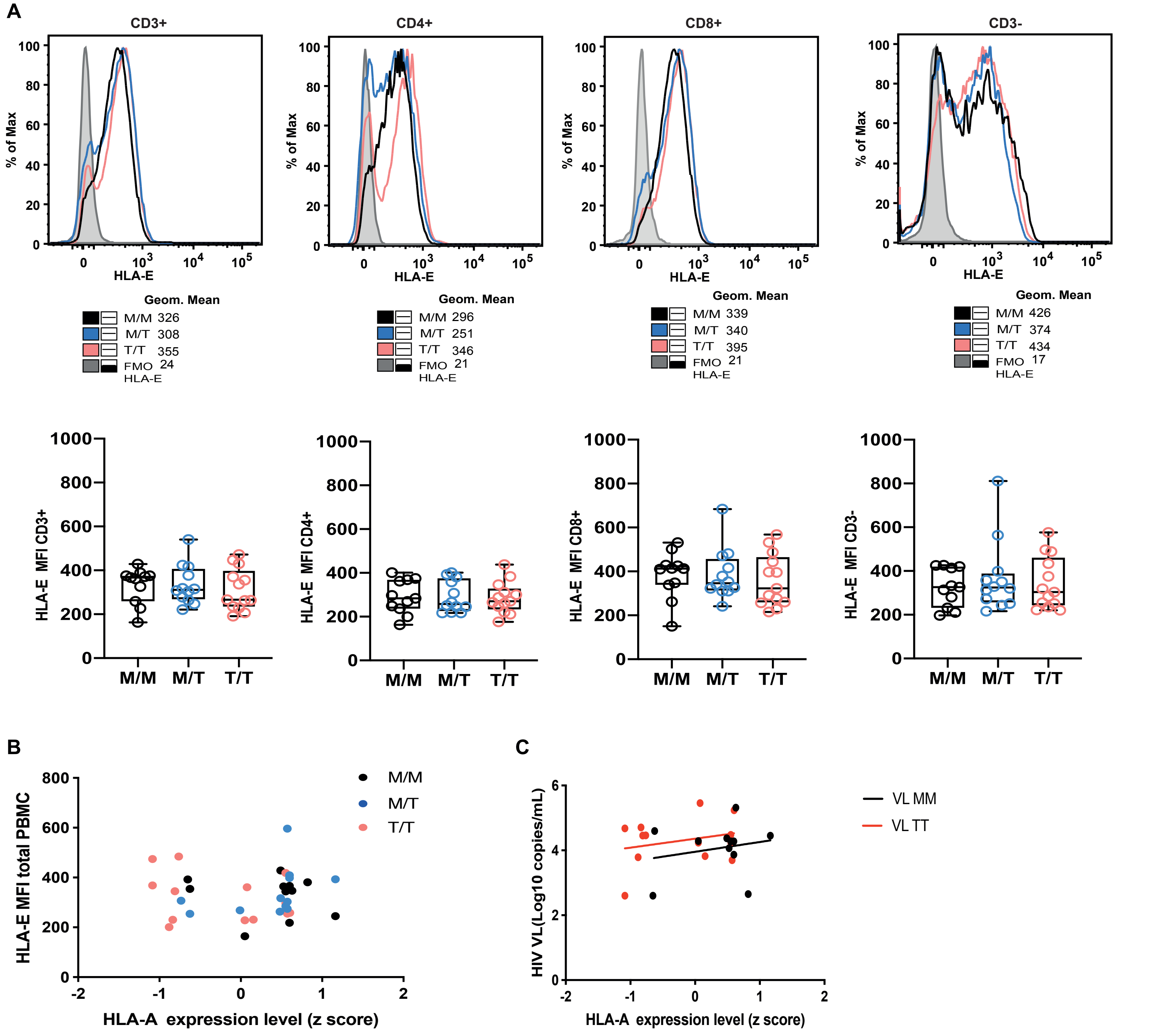

S1. Suppl. Figure S1. The effect of HLA-A expression on surface HLA-E expression and HIV viral load (VL) by HLA-B -21 variant. (A) Representative histograms showing HLA-E expression on CD3+, CD4+, CD8+ and CD3- cells between groups as well as fluorescence minus one (FMO) control staining, and summary boxplots.
Each symbol represents data from an individual subject, boxes indicate median and quartiles and whiskers indicate the total range. (B) Surface expression levels of HLA-E (MFI) on total PBMC, according to HLA-A (z score) and HLA-B dimorphism in the study cohort. (C) Correlation of HLA-A expression levels (z score) with HIV VL in HLA-B -21M/M and T/T donors.
