# Supplementary material for: Subordinate Effect of -21M HLA-B Dimorphism on NK Cell Repertoire Diversity and Function in HIV-1 Infected Individuals of African Origin
Source: Front Immunol. 2020 Feb 18;11:156. doi: 10.3389/fimmu.2020.00156 (PMC7041644; doi:10.3389/fimmu.2020.00156)
Supplement: Supplementary file 4 [file Image_2.pdf]

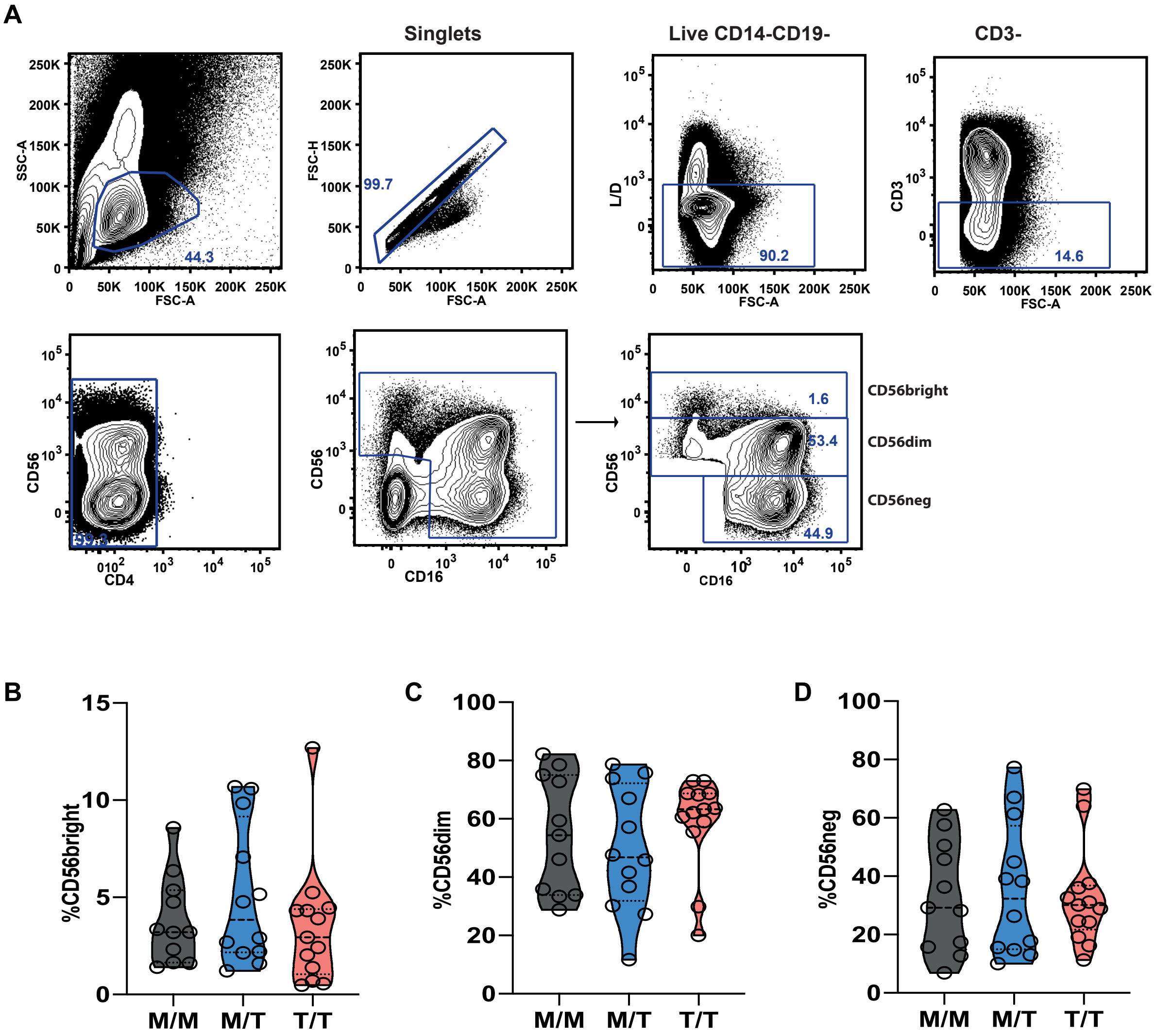

S2. Suppl. Figure S2. NK cell subset redistribution. (A) Representative example showing NK cells gated on live CD3-CD14-CD19-CD4- and subsets identified on the basis of CD56 and CD16 expression. Summary box and violin plots of the frequencies of (B) CD56bright, (C) CD56dim and (D) CD56neg NK cell subsets among M/M, M/T and T/T donors. Median and interquartile range is shown.
