# Supplementary material for: Subordinate Effect of -21M HLA-B Dimorphism on NK Cell Repertoire Diversity and Function in HIV-1 Infected Individuals of African Origin
Source: Front Immunol. 2020 Feb 18;11:156. doi: 10.3389/fimmu.2020.00156 (PMC7041644; doi:10.3389/fimmu.2020.00156)
Supplement: Supplementary file 5 [file Image_3.pdf]

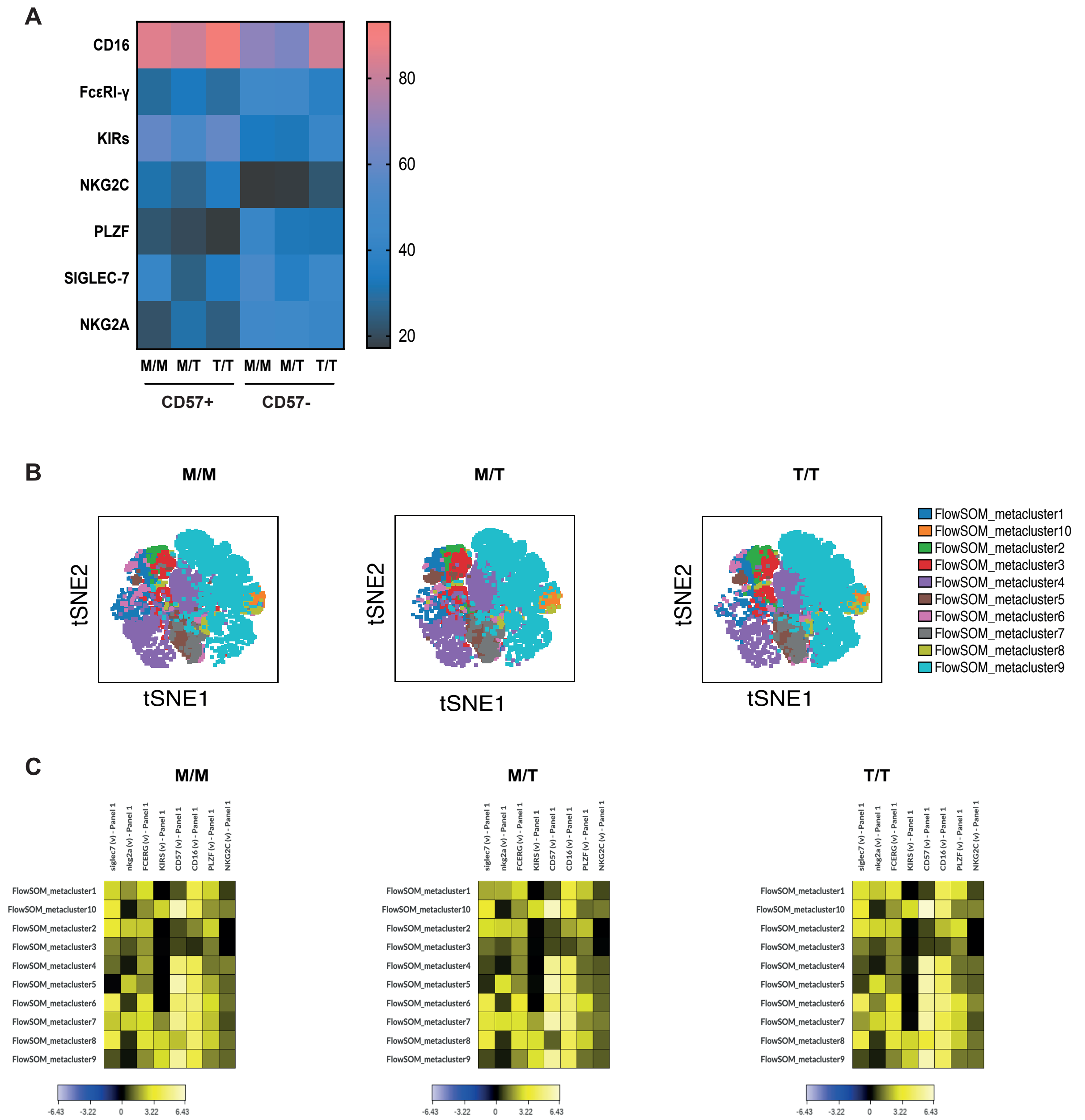

S3. Suppl. Figure S3 (A) Heat map representation of the mean proportion of expression of the markers as shown within the CD56dim CD57+ and CD57- fractions in M/M, M/T and T/T subjects. (B) Flow Self-Organising Maps (FlowSOM) clustering analysis of NK cells from compiled donors in each study group and identified metacluster populations overlaid on viSNE maps. (C) Heatmap view of the FlowSOM metaclusters by clustering channel to identify the phenotype of each metacluster. For heatmap visualisation data were scaled with transformed values based on the transformed ratio of the minimum value in the heatmap compared to each datapoint within the heatmap.
